# Robust formation of amorphous Sb_2_S_3_ on functionalized graphene for high-performance optoelectronic devices in the cyan-gap

**DOI:** 10.1038/s41598-020-70879-1

**Published:** 2020-09-10

**Authors:** Ju-Hung Chen, Sheng-Kuei Chiu, Jin-De Luo, Shu-Yu Huang, Hsiang-An Ting, Mario Hofmann, Ya-Ping Hsieh, Chu-Chi Ting

**Affiliations:** 1 Graduate Institute of Opto-Mechatronics, Department of Mechanical Engineering, National Chung Cheng University, Chia-Yi, 62102 Taiwan, R.O.C.; 2 Institute of Atomic and Molecular Sciences, Academia Sinica, Taipei, 10617 Taiwan, R.O.C.; 3 Department of Mechanical Engineering, National Chiao Tung University, Hsin-Chu, 30010 Taiwan, R.O.C.; 4 Department of Physics, National Taiwan University, Taipei, 10617 Taiwan, R.O.C.; 5 Advanced Institute of Manufacturing With High-Tech Innovations, National Chung Cheng University, Chia-Yi, 62102 Taiwan, R.O.C.; 6 Department of Materials Science and Engineering, Feng Chia University, Taichung, 40724 Taiwan, R.O.C.

**Keywords:** Chemistry, Energy science and technology, Engineering, Materials science, Nanoscience and technology, Optics and photonics, Physics

## Abstract

Despite significant progress in the fabrication and application of semiconductor materials for optical emitters and sensors, few materials can cover the cyan-gap between 450 and 500 nm. We here introduce a robust and facile method to deposit amorphous Sb_2_S_3_ films with a bandgap of 2.8 eV. By exploiting the tunable functionality of graphene, a two-dimensional material, efficient deposition from a chemical was achieved. Ozone-generated defects in the graphene were shown to be required to enhance the morphology and quality of the material and comprehensive characterization of the seed layer and the Sb_2_S_3_ film were applied to design an optimal deposition process. The resulting material exhibits efficient carrier transport and high photodetector performance as evidenced by unprecedented responsivity and detectivity in semiconductor/graphene/glass vertical heterostructures. (112 A/W, 2.01 × 10^12^ Jones, respectively).

## Introduction

Solid-state light emitters have improved the efficiency, availability, and comfort of lighting in our daily lives. Through a combination of red, green and blue emissions white light can be generated but the quality of color-rendering is not good enough due to the missing cyan emission around 450–500 nm, which is also called the cyan gap^1–3^. To fulfill the missing cyan emission component in the PL spectrum, it is urgent to find a semiconductor material with the bandgap at 2.8 eV that can fill the cyan gap presents in the high-quality white light. GaN possesses a bandgap at 2.8 eV but also has a 3.27 eV bandgap for the ultraviolet emission^4^. Zincblende ZnSs also has a 2.8 eV bandgap structure, but it needs to be under 10 K environment to adjust the bandgap from 2.7 to 2.8 eV^4^. MnS is another wide-gap semiconductor when its structure is rocksalt configuration, but the bandgap energy is a range of 2.8–3.2 eV^5^. Semiconductors mentions above have the necessary bandgap at 2.8 eV, but none of them have a good absorption coefficient in the cyan gap.

In chalcogenide semiconductors, Sb_2_S_3_ is a promising semiconductor material with different phase structures (a crystal phase called antimony trisulfide or stibnite, an amorphous phase called metastibnite) depending on the process. Whereas the stibnite phase has a direct energy gap ranging from 1.7 to 1.8 eV, the amorphous phase has a direct energy gap ranging from 1.7 to 2.8 eV^6,7,8^. In the amorphous phase, it has a very high absorption coefficient (1.8 × 10^5^ cm^−1^)^9^ at a wavelength of 450 nm. Different from many other semiconductors, Sb_2_S_3_ films can be deposited by a variety of facile, inexpensive and scalable methods such as thermal evaporation method^10^, sputtering method^11^, polyol reflux method^12^, chemical vapor deposition method^13^, spray-lysis method^14^, hydrothermal method^15^, and chemical bath deposition method^16–19^. However, the formation of amorphous material with stable band-gap properties remains elusive.

We here demonstrate the robust deposition of amorphous Sb_2_S_3_ from a chemical bath by utilizing functionalized graphene as a substrate. Since graphene has a poor lattice mismatch with crystalline Sb_2_S_3_, only amorphous Sb_2_S_3_ can be deposited on functionalized graphene^20,21^. To increase the efficiency of deposition, UV ozone irradiation is utilized to functionalize the surface of graphene. Upon optimized deposition, the amorphous Sb_2_S_3_ exhibits high morphological stability and good electrical properties. Optoelectronic characterization reveals a bandgap of 2.85 eV and high sensitivity to blue light irradiation yielding high photo-responsivity values (112 A/W), and detection rate (2.01 × 1,012 Jones). The presented robust and scalable deposition of Sb_2_S_3_ on graphene (SSG) opens up new routes for large bandgap optoelectronic devices in light emission and solar cells.

## Methods

Graphene was grown on untreated Copper foil (JX corp., 99.9% purity, 35 um thickness) following previous reports^22^. The growth pressure was kept constant at 10 Torr and the flow rate ratio of H2/CH4 was fixed at 20 resulting in identical partial pressures for various flow speeds. A standard wet-chemical transfer process was employed to remove graphene from the growth substrate and deposit it on a 300 nm Si/SiO_2_ substrate for further characterization^23^.

Sb_2_S_3_ film was made from the literature published by PK Nair et al. in 1997^22^. We used sodium thiosulfate (Na_2_S_2_O_3_·5H2O) and antimony trichloride (SbCl_3_) as the source of sulfur ions and the source of cerium ions when antimony trichloride (SbCl_3_) is reacted with sodium thiosulfate (Na_2_S_2_O_3_) and deionized water (DI Water). Absorbance, A%, and Transmittance, T% were measured by Thermo Scientific UV–Vis spectrophotometer, I–V and I–T measurements were conducted in Keithley 2636B on macroscopic (1 cm^2^) samples using silver paint electrodes. Raman spectroscopy was carried out in a homebuilt confocal setup using a 532 nm excitation and PL spectra were measured in Acton Series (SP-2156) spectroscopy. X-ray diffraction analysis was carried out in BRUKER D2 PHASER X-ray diffractometer. SEM images were carried out in Hitachi S-480 Scanning Electron Microscope.

## Results and discussion

**Figure 1:**
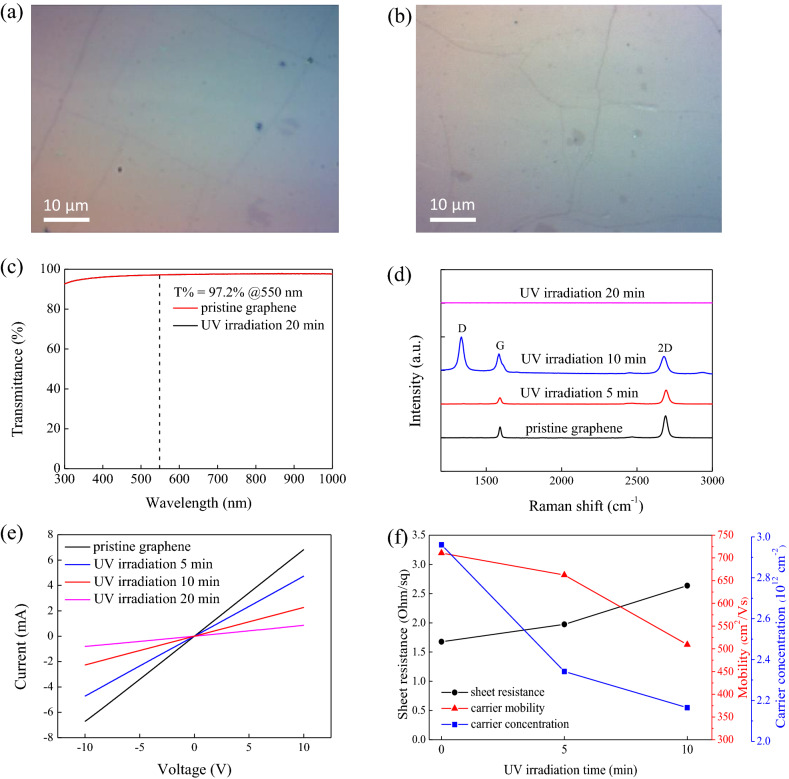
Before (**a**) and after (**b**) graphene UV irradiation OM diagram at 100 ×, scale bar: 10 μm. **(c**) Transmittance spectrum of graphene without and with UV irradiation for 20 min. (**d**) Graphene Raman spectra showing unmodified and UV ozone irradiation time of 5, 10, 20 min. (**e**) IV curves of graphene for unmodified and UV irradiation at 5, 10, 20 min, respectively. (**f**) Sheet resistance, carrier mobility, and carrier concentration of graphene for unmodified and UV irradiation at 5, 10, 20 min, respectively.

It can be seen that the surface morphology of graphene before and after UV irradiation is not much different in Figs. 1a and 1b, and the boundary defects of graphene are not increased, resulting in no significant decrease in the transmittance. At a wavelength of 550 nm, the light transmittance is about 97.2% which is close to the single-layer graphene in the literature as shown in Fig. 1c, and surface oxidation of graphene does not affect the light transmittance of graphene after UV irradiation.

Raman spectroscopy is applied to confirm single-layer graphene property after UV irradiation. A low ratio of I_D_/I_G_ and the high I_2D_/I_G_ ratio of unmodified graphene (0.05 and 1.63, respectively) corroborate the presence of single-layer graphene with few defects (shown as in Fig. S2). After 5 and 10 min of UV irradiation, the I_D_/I_G_ peak ratios increased to 0.91 and 1.8, respectively. The UV irradiation for 20 min showed D, G, and 2D band signals without graphene. Therefore, the longer the UV-irradiation time is applied, the more carbon–carbon sp2 bonding structures will be destroyed in graphene, resulting in a larger ID/IG peak ratio and even disappeared eventually, as shown in Fig. 1d.

From I–V sweep with a bias voltage of − 10 to 10 V in Fig. 1e, the resistance of graphene is proportional to UV irradiation time and a current drop from 6.82 to 2.27 mA at a voltage of 10 V after graphene is UV irradiated for 20 min is also observed in Fig. 1f. Hall effect measurement results showed that the sheet resistance of graphene after 10 min irradiation increased from 1,676 to 2,636 Ω/sq, and the carrier mobility was reduced from 710 to 509 ${\text{ cm}}^{2} /{\text{V}}\,{\text{s }}$. The graphene after 20 min of UV irradiation could not measure the sheet resistance and carrier mobility due to its high resistance. We hypothesize that the high-reactive oxygen atom breaks the carbon–carbon sp2 bond and π-bond structure to form the sp3 hybrid orbital, producing oxygen functional groups (COC, C–OH, and CH). The longer the UV irradiation time, the more oxygen functional groups (C–O–C, C–OH, and C–H) will be formed, so the resistance will increase as the UV irradiation time increases^24,25^.

**Figure 2:**
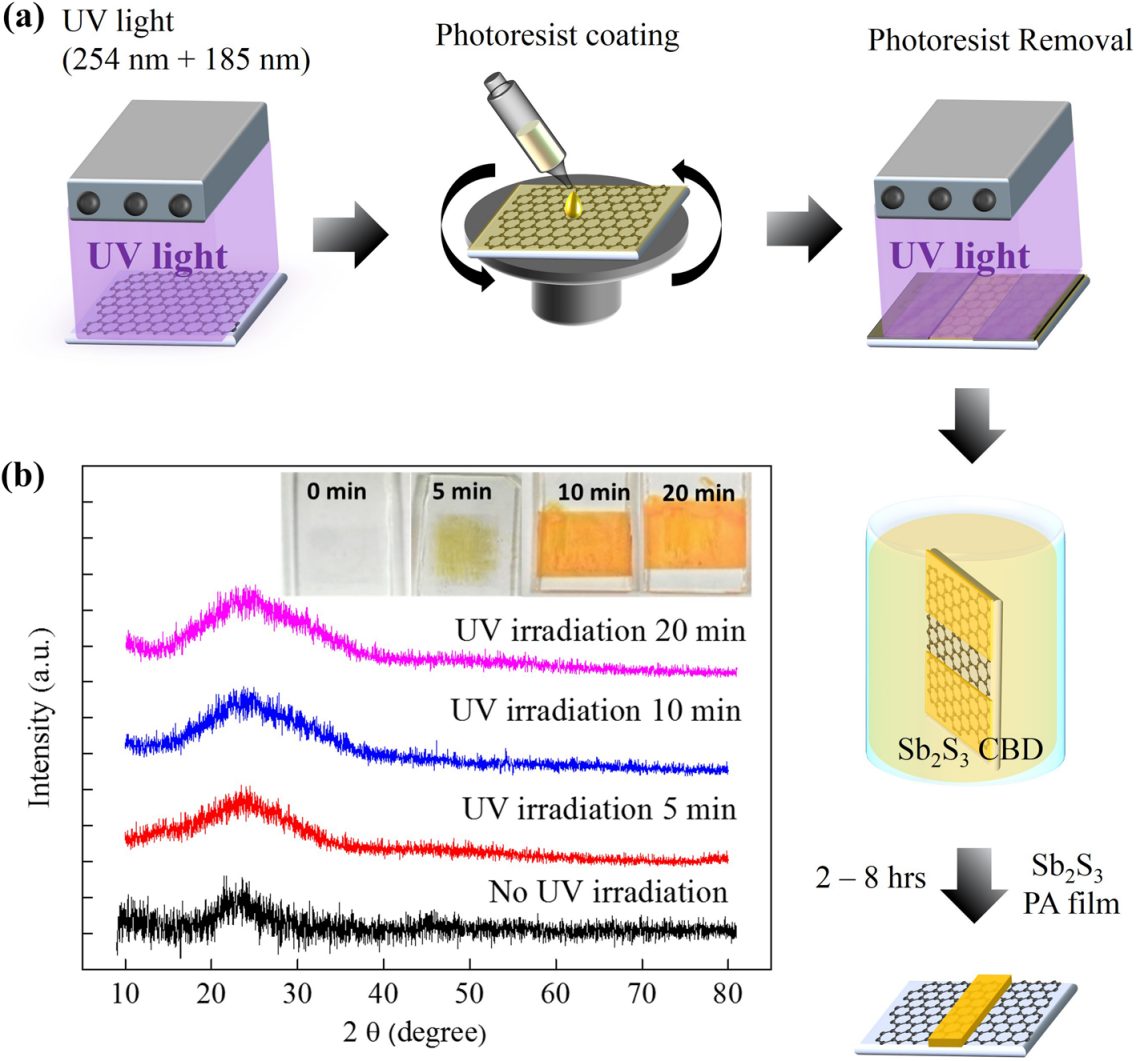
(**a**) A schematic diagram showing the process of deposition of Sb_2_S_3_ film on functionalized graphene after photolithography. (**b**) The XRD patterns of the Sb_2_S_3_ deposited on the graphene without and with a UV irradiation time of 5, 10, 20 min by 4 h chemical bath deposition time.

The UV-irradiated graphene-based plate was vertically fixed and immersed in a beaker of Sb_2_S_3_ solution after photolithography, and the beaker was immersed in a low-temperature water bath and the temperature was maintained below 10 °C. The Sb_2_S_3_ thin-film deposition time was conducted for 2, 4, 6, 8 h per batch. When the plate was taken out, the color of the film on the plate has become orange. Detail synthesis parameters and reactions of Sb_2_S_3_ synthesis are schematically shown in Fig. 2a and described the supplemental information.

We observed that Sb_2_S_3_ cannot be effectively deposited on pristine graphene, but with the increase of graphene UV ozone irradiation time, Sb_2_S_3_ can be deposited on graphene more effectively in Fig. 2b. A pale yellow color was observed from Sb_2_S_3_ film when the UV irradiation time reaches 5 min, but Sb_2_S_3_ is amorphous according to XRD pattern. After 10 min and 20 min of UV irradiation time, it was found that the color of the Sb_2_S_3_ film deposited on the graphene became dark orange, indicating that the Sb2S3 film was thicker, but the XRD patterns were still amorphous for thicker Sb_2_S_3_ film.

After UV irradiation of graphene, C–OH and C–O–C bonds are formed on the surface. These bonds may be negatively charged and can be used to help adsorb Sb_2_S_3_ in the CBD reaction. The longer the UV irradiation time, the more C–OH and C–O–C bonds are formed, which causes the graphene to be oxidized and deteriorated, but it can help to adsorb more Sb_2_S_3_ molecules and increase Sb_2_S_3_ film thickness^24^.

Photo-responsivity time measurements were examined to find the appropriate UV irradiation time for the highest photocurrent at 4 h CBD Sb_2_S_3_ film growth condition. It shows that the components with the modified 5 min and 10 min have a brighter photocurrent increased after UV irradiation, but the photocurrent of the component modified for 20 min has almost no rise as shown in Fig. S3b. Therefore, 10 min UV irradiation for graphene to produce functionalization is the fixed-parameter for different CBD Sb_2_S_3_ film growth times.

**Figure 3:**
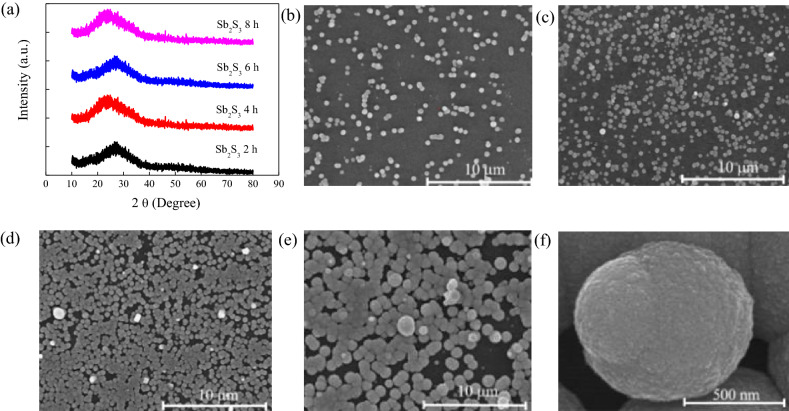
(**a**) The XRD patterns of 10 min UV irradiation time of graphene with 2, 4, 6, and 8 h Sb_2_S_3_ deposition time, respectively. (**b**–**e**) SEM top view of Sb_2_S_3_ film at 2, 4, 6, and 8 h CBD growth time, respectively. (**f**) A single particle of Sb_2_S_3_ for 8 h CBD growth time.

Not only UV irradiation time affect photocurrent and photo-responsivity time, but the thickness of Sb_2_S_3_ film is also another key parameter to improve photoelectronic properties. It can be found that the longer the deposition time, the thicker the Sb_2_S_3_ film but the amorphous nature is retained from the XRD measurement in Fig. 3a. Scanning electron microscopy (SEM) was used to observe the Sb_2_S_3_ film deposited on the UV irradiated graphene for 2, 4, 6, and 8 h, respectively. The SEM cross-section of the Sb_2_S_3_ is shown for different growth times. It can be seen that the film thickness is 141 nm, 485 nm, 559 nm, and 832 nm at deposition time of 2–8 h. It can be seen that the longer the deposition time, the more Sb_2_S_3_ particles (Fig. 3f) aggregate together in 2D direction on the graphene and gradually form a film as shown in Fig. 3b–e.

**Figure 4:**
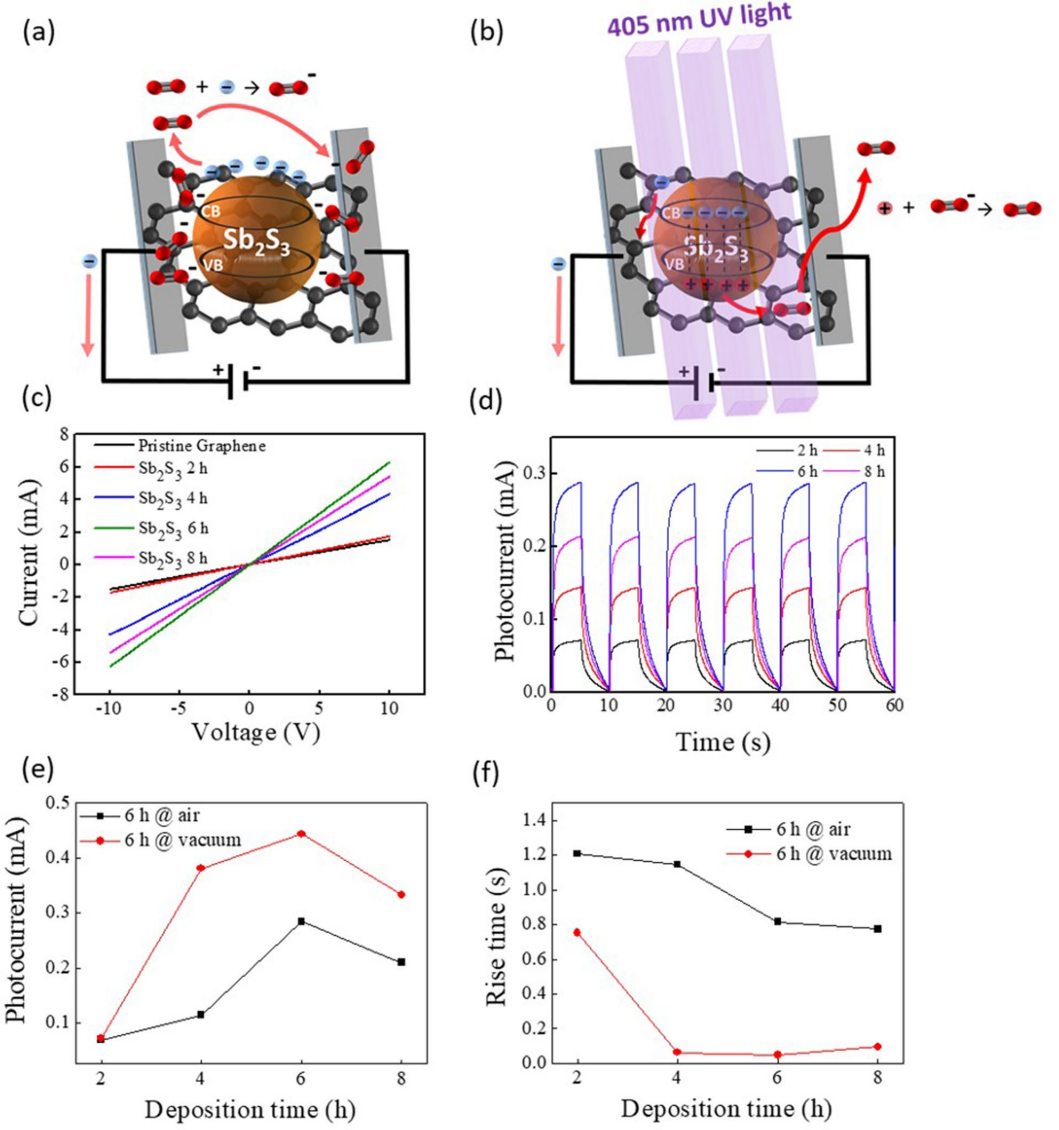
A schematic diagram showing carrier transport of SSG unilluminated (**a**) and illuminated (**b**) by 405 nm lasers, red-colored balls indicates oxygen atoms. (**c**) The I–V curves of SSG photodetector for 2, 4, 6 and 8 h Sb_2_S_3_ deposition time, respectively. (**d**) The I–T curves measurements of Sb_2_S_3_ at different Sb_2_S_3_ thin film growth times on graphene for atmosphere photocurrent, and rise time comparison in 2, 4, 6 and 8 h Sb_2_S_3_ deposition time of atmosphere (**e**) and vacuum (**f**), respectively.

The dimensions of the components are glass (1.5 cm × 1.5 cm), graphene (1 cm × 1 cm) and Sb_2_S_3_ (0.5 cm × 0.5 cm). The incident source of the excitation element is a 405 nm laser. The incident light intensity is 10–60 mW/cm^2^ and the measurement voltage is 1–15 V. The SSG composite components grown for 2–8 h were compared by 405 nm illumination I–V measurement, and it was found that the illumination current was significantly improved at a growth time of 6 h in Fig. 4c, compared to 2, 4, and 8 h CBD deposition time. The I–T curves of SSG at different growth times for atmosphere and vacuum conditions are shown separately in Fig. 4d. At a fixed bias voltage of 15 V, we measured the photo-responsivity of the SSG device at 2, 4, 6 and 8 h in the atmosphere and vacuum environment respectively. Since a higher photocurrent is observed at Sb_2_S_3_ film on the UV irradiated graphene due to the oxygen functional group on the graphene surface, the graphene defects are increased, and the graphene high carrier mobility is sacrificed^26^. Therefore when an electron–hole pair is generated after illumination, the photocarrier is trapped by the modified graphene defect before being injected into the pure graphene layer, compared to no illumination condition, as schematically shown in Fig. 4a,b, resulting in a decrease in carrier mobility, which makes it impossible to obtained photocurrent in a short time, causing the dark current to rise to the illumination current.

A comparison of the signal peak, photocurrent and rise time of the atmosphere and vacuum in Fig. 4e,f. Due to the oxygen atom is reduced in the vacuum environment, the formed depletion region is narrower, and the carrier concentration is increased. Therefore, the photocurrent under vacuum is higher than the atmosphere, and the rise time is also much shortened. The photo-responsivity characteristics of this device are the same as those of photodetection device fabricated by rGO and chemical functionalization, because of some defects in the film cause long rise and fall times^27^.

When unilluminated, part of the photocarriers are still trapped by the modified graphene defects, making the photocarriers difficult to recombine and prolonging the excitons recombination time, cause additional a few seconds needed for the rise and fall times to complete the on/off action in the SSG.

A large amount of photocurrent can further increase the post-illumination reaction of graphene. But not all of the electrons and holes that are generated are pulled away by the applied electric field because there are many oxygen atoms (O_2_) in the atmosphere that will attract electrons that jump to high energy states, producing negatively charged oxygen atoms (${\text{O}}_{2}^{ - }$). The above reasons will cause the rise time to be lower by this effect.

In this experiment, it was found that due to the increase in the thickness of the Sb_2_S_3_ film and the unevenness of the surface, the trap inside the film was relatively increased, so that the carrier was not composited before being transferred to the graphene^28^. Therefore, the growth time of 6 h of illumination I–V and the atmospheric and I–T under vacuum have the best response, so the subsequent experiments are based on the best growth parameters of 6 h as the benchmark.

Different voltages were used to explore the optimal parameters of SSG components. In the experiment, a 405 nm laser light intensity of 60 mW and a switching time of 5 s was used to irradiate the SSG via 6 h CBD Sb_2_S_3_ film deposition time. When the applied voltage became smaller, the generated photocurrent decreased, and the electric field inside the material is reduced. Therefore, the internal recombination ability increases for the electron–hole generated in Sb_2_S_3_ film under a small electric field, so the photocurrent is reduced. If the applied voltage is too large, it will produce an overshoot phenomenon. When the applied voltage is 15 V, the component has the best photocurrent of 6.18 × 10^–4^ A and the rise time of 27.05 ms (Fig. S5a).

A vacuum I–T plot of the different light intensity of the SSG component is shown in Fig. S5b. Change the different light intensities to explore the characteristics of the best parameters of SSG components. Using a 405 nm Laser to illuminate the 6 h CBD deposition SSG, the component is fixed at a voltage of 15 V, the switching time is 5 s, and the light intensity is from 10 to 60 mW. It can be found that when the light intensity is weakened, the current also drops. The higher the incident light intensity, the more photons can excite the Sb_2_S_3_ to generate more electron–hole pairs, so the photocurrent increases. When the weaker light is applied, since only the surface of the Sb_2_S_3_ generates a carrier, the surface carrier is transferred to the graphene, and the photocurrent is reduced by the composite of the material and the graphene defect.

The photocurrent increases as the light intensity increases^29,30^. The intrinsic defect density of the device can be found through the relationship between photocurrent and light intensity measured by different light intensities, which can be derived from the linear fitting Power Law.

1 $${\text{I}} \propto {\alpha P}^{\beta }$$

I is the photocurrent measured by the component at 15 V, α is the proportionality constant, P is the optical power intensity of 405 nm, and β represents the defect density of the component itself. When the component defect density is extremely small, the photocurrent is linearly proportional to the incident light intensity, θ should be close to 1, conversely. If the component defect density is extremely large, θ should be much smaller than 1.

**Figure 5:**
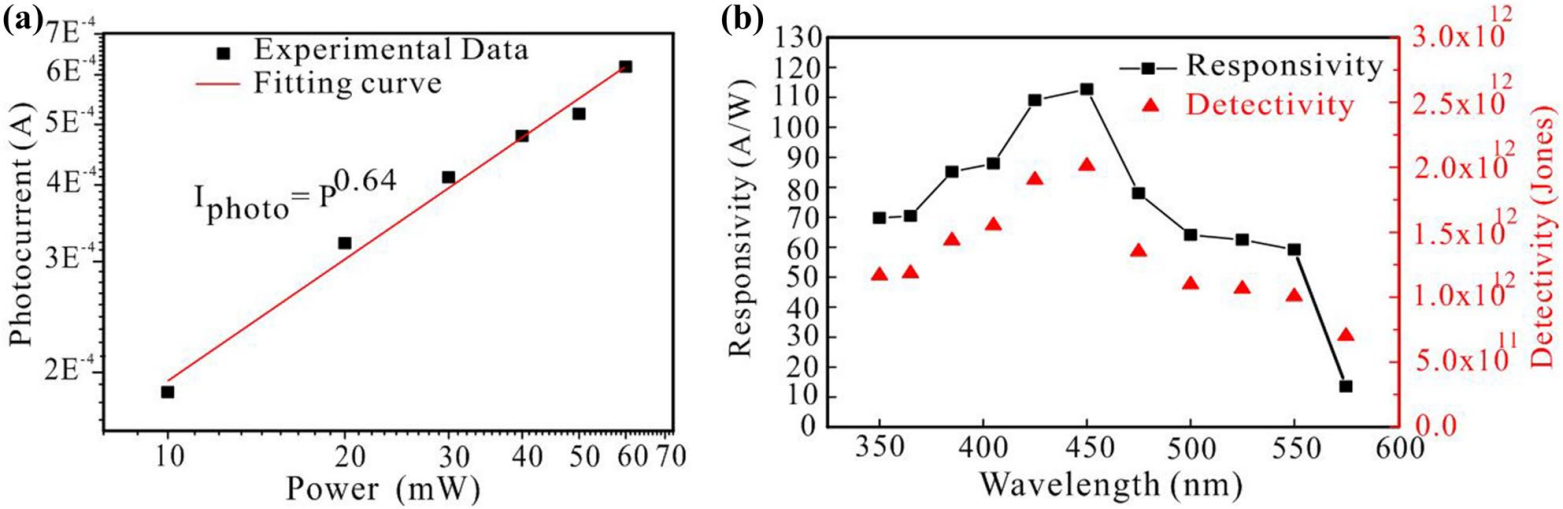
(**a**) The I-P curve measurement of the SSG device illuminated by the 405 nm laser at different laser powers. (**b**) Photo-responsivity of SSG at 15 V under 405 nm laser.

The photoelectric response results of a 405 nm laser light illuminating element with a power density ranging from 10 to 60 mW, and we use the fitting I–P to get a beta value of 0.64 (Fig. 5a). Photo-responsivity (R) is a key parameter for evaluating the performance of a photodetector. It is defined as the photocurrent generated by the incident light per unit of power on the effective area of the device. R can be calculated by using the following formula.

2 $${\text{R}} = {\text{I}}/{\text{P}} \cdot {\text{S}}$$

I is the photocurrent (lighting current–dark current), P is the incident light intensity of the xenon lamp, and S is the effective illumination area. Because our growing material area is larger than the illumination spot area, we do not need the area item in this experiment.

When the light of the wavelength of 450 nm is utilized, the optical response is 112 A/W when the optical power intensity is 1 μW/cm^2^. In the ultraviolet band, when the light of the wavelength of 350 nm is irradiated. The optical response of the component is also 69.76 AW^−1^. When the wavelength of light absorbed by Sb_2_S_3_ exceeds 568 nm, the photo-responsivity value immediately drops to zero and there is no absorption at all. Therefore, it can be found that Sb_2_S_3_ is only in the ultraviolet to the visible range for photo-responsivity to occur, and the best photo-responsivity value in the visible light band will also confirm the argument that the Sb_2_S_3_ enthalpy gap deposited in this study is about 2.2 eV. The detection rate corresponding to the response value can be calculated by the following formula.

3 $${\text{D}}\uplambda* = {\text{ R}}S^{\frac{1}{2}} /(2e{\text{Id}}){1}/{2}$$

R is the spectral response value, Id is the dark current measured by Xenon lamp, S is the effective illumination area, and e is 1.609 × 10^–19^ C. After calculation by the above formula, it has the highest response value at 450 nm and the highest detection rate, which is 2.01 × 10^12^ Jones, shown as Fig. 5b.

## Conclusion

Using graphene with different UV irradiation times to find the best growth parameters, we found that Sb_2_S_3_ can be deposited on graphene after UV irradiation for 5, 10, and 20minutes. In the measurement of photoelectric characteristics, no photocurrent and switching characteristics were found from 5 and 20 min of UV irradiation. Therefore, in this study, the modified 10-min graphene was used as the best parameter for Sb_2_S_3_ film deposition. The effects of different Sb_2_S_3_ film deposition time from 2 to 8 h on the photoelectric properties of graphene were compared. It was found that the film thickness was not proportional to the photocurrent increase. Due to the increase in film thickness, the charge was transferred during the transfer process due to the graphene defect trap produced via UV irradiation. Influenced by the scattering between the particles, thus increasing the chance of carrier recombination and slowing down the rise time. Finally, the UV ozone irradiation was modified for 10 min and after 6 h of Sb_2_S_3_ film deposition, which had the best rise time of 27.05 ms for the 405 nm laser. Optimal photo-responsivity value 112 A/W and detection rate 2.01 × 10^12^ Jones, demonstrating that semiconductor Sb_2_S_3_ materials can increase the photo-responsivity of graphene.

## Supplementary information

Supplementary information.

## References

[1] Fang MH (2016). Enhance color rendering index via full spectrum employing the important key of cyan phosphor. ACS Appl. Mater. Interfaces.

[2] Liu Y (2017). An excellent cyan-emitting orthosilicate phosphor for NUV-pumped white LED application. J. Mater. Chem. C.

[3] Zhao M (2019). Emerging ultra-narrow-band cyan-emitting phosphor for white LEDs with enhanced color rendition. Light Sci. Appl..

[4] Shahedipour F, Wessels BW (2000). Investigation of the formation of the 2.8 eV luminescence band in p-type GaN: Mg. Appl. Phys. Lett..

[5] Lokhande CD (1998). Process and characterisation of chemical bath deposited manganese sulphide (MnS) thin films. Thin Solid Films.

[6] Salem AM, Selim MS, Salem AM (2000). Structure and optical properties of chemically deposited Sb2S3thin films. J. Phys. D Appl. Phys..

[7] Liu M, Gong Y, Li Z, Dou M, Wang F (2016). A green and facile hydrothermal approach for the synthesis of high-quality semi-conducting Sb2S3 thin films. Appl. Surf. Sci..

[8] Maghraoui-Meherzi H, Ben Nasr T, Kamoun N, Dachraoui M (2010). Structural, morphology and optical properties of chemically deposited Sb2S3 thin films. Phys. B Condens. Matter.

[9] Versavel MY, Haber JA (2007). Structural and optical properties of amorphous and crystalline antimony sulfide thin-films. Thin Solid Films.

[10] Zhang D (2012). Understanding charge transfer at pbs-decorated graphene surfaces toward a tunable photosensor. Adv. Mater..

[11] Medina-Montes M, Montiel-González Z, Mathews NR, Mathew X (2017). The influence of film deposition temperature on the subsequent post-annealing and crystallization of sputtered Sb2S3 thin films. J. Phys. Chem. Solids.

[12] Chao J (2014). Synthesis of Sb2S3 nanowall arrays for high performance visible light photodetectors. Mater. Res. Bull..

[13] Castro JR, Dale P, Mahon MF, Molloy KC, Peter LM (2007). Deposition of antimony sulfide thin films from single-source antimony thiolate precursors. Chem. Mater..

[14] Gadakh S, Bhosale C (2003). Effect of concentration of complexing agent (tartaric acid) on the properties of spray deposited Sb2S3 thin films. Mater. Chem. Phys..

[15] Li C, Yang X, Liu Y, Zhao Z, Qian Y (2003). Growth of crystalline Sb2S3 nanorods by hydrothermal method. J. Cryst. Growth.

[16] Mane R, Lokhande C (2000). Chemical deposition method for metal chalcogenide thin films. Mater. Chem. Phys..

[17] Wang Z (2017). Low-cost TiO2/Sb2 (S, Se) 3 heterojunction thin film solar cell fabricated by sol-gel and chemical bath deposition. Mater. Sci. Semicond. Process..

[18] Mushtaq S, Ismail B, Zeb MA, Kissinger NS, Zeb A (2015). Low-temperature synthesis and characterization of Sn-doped Sb2S3 thin film for solar cell applications. J. Alloy. Compd..

[19] Krishnan B, Arato A, Cardenas E, Roy TD, Castillo G (2008). On the structure, morphology, and optical properties of chemical bath deposited Sb2S3 thin films. Appl. Surf. Sci..

[20] Alemi A (2011). Hydrothermal synthesis of Sb2S3 nanorods using iodine via redox mechanism. J. Nanomater..

[21] Karpan VM (2007). Graphite and graphene as perfect spin filters. Phys. Rev. Lett..

[22] Nair RR (2008). Fine structure constant defines visual transparency of graphene. Science.

[23] Güneş F (2011). UV-light-assisted oxidative sp3 hybridization of graphene. NANO.

[24] Choi MS, Lee SH, Yoo WJ (2011). Plasma treatments to improve metal contacts in graphene field effect transistor. J. Appl. Phys..

[25] Hasan MT (2017). Optical band gap alteration of graphene oxide via ozone treatment. Sci. Rep..

[26] Ick Son D, Yeon Yang H, Whan Kim T, Il Park W (2013). Photoresponse mechanisms of ultraviolet photodetectors based on colloidal ZnO quantum dot-graphene nanocomposites. Appl. Phys. Lett..

[27] Li QH, Gao T, Wang YG, Wang TH (2005). Adsorption and desorption of oxygen probed from ZnO nanowire films by photocurrent measurements. Appl. Phys. Lett..

[28] Liang FX (2016). Highly sensitive UVA and violet photodetector based on single-layer graphene-TiO_2_ heterojunction. Opt. Express.

[29] Gong X (2009). High-detectivity polymer photodetectors with spectral response from 300 nm to 1450 nm. Science.

[30] Hsieh Y-P, Shih C-H, Chiu Y-J, Hofmann M (2016). High-throughput graphene synthesis in gapless stacks. Chem. Mater..

[31] Kim KS (2009). Large-scale pattern growth of graphene films for stretchable transparent electrodes. Nature.

