# Supplementary material for: Robust formation of amorphous Sb2S3 on functionalized graphene for high-performance optoelectronic devices in the cyan-gap
Source: Sci Rep. 2020 Sep 10;10:14873. doi: 10.1038/s41598-020-70879-1 (PMC7483733; doi:10.1038/s41598-020-70879-1)
Supplement: Supplementary file 1 — Supplementary information. [file 41598_2020_70879_MOESM1_ESM.docx]

**Supplementary Information**

**Robust formation of amorphous Sb_2_S_3_ on functionalized graphene for high-performance optoelectronic devices in the cyan-gap**

Ju-Hung Chen^1‡^, Sheng-Kuei Chiu^2‡^, Jin-De Luo^1^, Shu-Yu Huang^1^, Hsiang-An Ting^3^, Mario Hofmann^4^, Ya-Ping Hsieh^2^, Chu-Chi Ting^1,5^*

1. Institute of Opto-Mechatronics, Department of Mechanical Engineering, National Chung Cheng University, Chia-Yi, Taiwan 62102

2. Institute of Atomic and Molecular Sciences, Academia Sinica, Taipei, 10617, Taiwan

3. Department of Mechanical Engineering, National Chiao Tung University, Hsin-Chu, 30010, Taiwan

4. Department of Physics, National Taiwan University, Taipei 10617, Taiwan

5. Advanced Institute for Manufacturing with High-Tech Innovations, National Chung Cheng University, Chia-Yi, 62102, Taiwan

‡These authors contributed equally

*Correspondence should be addressed to C.T. (ccting@ccu.edu.tw)

**Graphene Synthesis and Lithography**

The graphene used in this study was synthesized follow by Hsieh’s work.^22^ The unpolished and polished copper foil is placed in a quartz tube, hydrogen is used as a catalyst and methane is used as a carbon source, and is thermally cracked and deposited on a copper foil to form a multilayer or single-layer graphene.

To accurately control the position and channel (0.5 cm * 0.5 cm) of the strontium sulfide film grown on graphene, we use the yellow lithography process as follows: first, apply the photoresist evenly to the transferred graphene component with a curler, and softly bake it to remove the solvent to cure the photoresist to increase adhesion. Second, fix the reticle on the photoresist and use a UV lamp to illuminate the position where the bismuth sulfide film grows. Third, use a developer to remove the photoresist at the channel location, rinse with deionized water, and finally dry with a nitrogen gun.

**Graphene Modification via UV Irradiation**

The exposed graphene/glass substrate is placed in a UV ozone-modified purifier consisting of 254 nm + 185 nm lamps and irradiated for 5 to 20 minutes to add more functional groups to the graphene surface and to increase hydrophilicity for more effective in depositing Sb_2_S_3_ on the surface of graphene.

**Sb_2_S_3_ Thin-film Precursor Solution Synthesis**

In this study, we made Sb_2_S_3_ from the literature published by PK Nair et al. in 1997. We used sodium thiosulfate (Na_2_S_2_O_3_·5H_2_O) and antimony trichloride (SbCl_3_) as the source of sulfur ions and the source of cerium ions, when antimony trichloride (SbCl_3_) is reacted with sodium thiosulfate (Na_2_S_2_O_3_) and deionized water (DI Water), the equation is as follows:

2SbCl_3_ + 3Na_2_S_2_O_3_ → Sb_2_ (S_2_O_3_) + 6NaCl (S1)

Sb_2_ (S_2_O_3_)3 + 6H_2_O → Sb_2_S_3_ + 3HS$O_{4}^{-}$ + 3H_3_O^+^ (S2)

At this time, Sb_2_ (S_2_O_3_)_3_ will produce Sb_3_^+^ and S_2_$O_{3}^{2-}$, where S_2_$O_{3}^{2-}$will hydrolyze to release sulfur ions, as follows

S_2_$O_{3}^{2-}$ + H_2_O ${}_{\to}^{\leftarrow}$S$O_{4}^{2-}$ + S^2-^ + 2H^+^ (S3)

Finally, the sulfide ion and the cerium ion react with each other to produce Sb_2_S_3_ film on the substrate, as follows:

2Sb_3_ + + 3S^2 -^ → Sb_2_S_3_ (S4)

3.75 g of Na_2_S_2_O_3_ and 20 g DI Water fully stirred with a magnet to clarify to solution A, 0.65 g SbCl_3_ was added to 2.5 ml acetone as solution B, then P solution was slowly poured into solution B at 10 °C, then 10 °C DI Water was slow. Slowly add to the mixed solution to make the solution reach 80 ml. After mixing, the solution will turn from white to light yellow and finally turn into orange-red to place the substrate into the solution.

**SSG Thin-film Synthesis via CBD Process**

The UV-ozone-modified graphene-based plate was vertically fixed and immersed in a beaker of Sb_2_S_3_ solution, and the beaker was immersed in a low-temperature water bath and the temperature was maintained below 10 °C. The Sb_2_S_3_ thin-film deposition time was taken for 2 hours per time. When the plate was taken out, the color of the film on the plate has become orange. The longer the deposition time, the darker orange color is, and the color of Sb_2_S_3_ solution will become clear. After a certain deposition time, the Sb^3+^ and S^2-^ ions in the Sb_2_S_3_ solution are completely deposited on the substrate, causing the solution to change from orange to clear and transparent until the Sb_2_S_3_ film can not be further to thicken.

**Graphene Structure and Photoelectric Characteristics Before and After UV Irradiation**

The Raman spectrum ID/IG peak ratios were 0.91 and 1.8, respectively, after 5 minutes and 10 minutes of UV-irradiation. The UV irradiation for 20 minutes showed D, G, and 2D band signals without graphene. Therefore, the longer the UV-irradiation time is applied, the more its surface structure will be destroyed in graphene, resulting in a larger ID/IG peak ratio and even disappeared eventually, as shown in Fig. S1.

**Figure S1** Graphene Raman spectroscopy I2D/IG and ID/IG ratio after UV ozone irradiation time of 5, 10, 20 minutes.

**Figure S2** (**a**) SSG Photodetector device I-V and I-T curves. (**b**) Photo-responsivity time measurements of different UV irradiation time of graphene (0, 5, 10, 20 mins) with 4 hours Sb_2_S_3_ deposition in the CBD process.

**SSG SEM analysis**

**Figure S3** (**a – d**) The SEM cross-section images of the Sb_2_S_3_ at deposition time of 2, 4, 6 and 8 hours, respectively.

**UV-Vis transmittance absorption spectrum analysis of SSG device**

From Fig. S3, it can be found that the thickness Sb_2_S_3_ deposition on graphene surface increases with the deposition time of Sb_2_S_3_, and the color gradually becomes darker, which is caused by the thicker Sb_2_S_3_ film deposited on the surface of graphene. In order to determine the absorption band of Sb_2_S_3_ on the graphene surface, the UV-visible absorption was measured. The penetration rate decreases as the film thickness increases. It is found in Fig. S4 (b) that the absorption intensity will increase significantly with the thickness of the Sb_2_S_3_ caused by the different deposition time. The absorption band of the SSG composite is about 300 ~ 450 nm from ultraviolet to visible. After the Sb_2_S_3_ deposited on the surface of graphene, it can be seen that the penetration rate and absorption rate of pure graphene and SSG have a large drop, which proves that Sb_2_S_3_ effectively increases the photo-responsivity of graphene.

Fig. S4. (c) shows that the value of the absorption spectrum of Fig. S4 (b) is calculated by Tauc-Sunds equation. The growth of the Sb_2_S_3_ film gap is about 2.2 eV, which is consistent with the literature.^31^

${(\alpha h\upsilon)}^{2}=A(h\upsilon-E_{g})$ (S5)

𝛼：Absorption coefficient, ℎ：Planck’s constant, A：constant, ℎ 𝜈：incidenc light energy, 𝐸𝑔：material energy gap, index 2: direct energy gap.

**Figure S4** The transmittance (**a**) and absorption spectra (**b**) of SSG for different Sb_2_S_3_ growth time. (**c**) Sb_2_S_3_ Photon energy diagram of ${\alpha h\upsilon}^{2}$.

**Figure S5** I-T curves of 6 hrs CBD deposition time of SSG at different voltages (**a**), and different laser powers (**b**) in high vacuum condition.

**Amorphous Sb_2_S_3_ film morphological stability observation by SEM**

6 hrs CBD growth time Sb_2_S_3_ film was examined the morphological stability as shown in Figure S6 a and b. The morphology of Sb­_2_S_3_ film after few months since the UV-irradiation experiment is not changed, and no collapsed Sb2S3 particles can be found which indicates our Sb_2_S_3_ film can last more than few months with good morphological stability.The resistance of Sb_2_S_3_ film can be risen due to broken Sb_2_S_3_ film, which causes by collapsed Sb_2_S_3_ particles. Again, no collapsed Sb_2_S_3_ particles can be found from Figure S6 b, which indicates our Sb_2_S_3_ film possesses good morphological stability. With this evidence, and consider the original surface roughness of Sb_2_S_3_ particle film which can present ±6 mA current performance at 10 V described in the main content, the resistance of our Sb_2_S_3_ film should not have major changes after few months since UV-irradiation experiments.


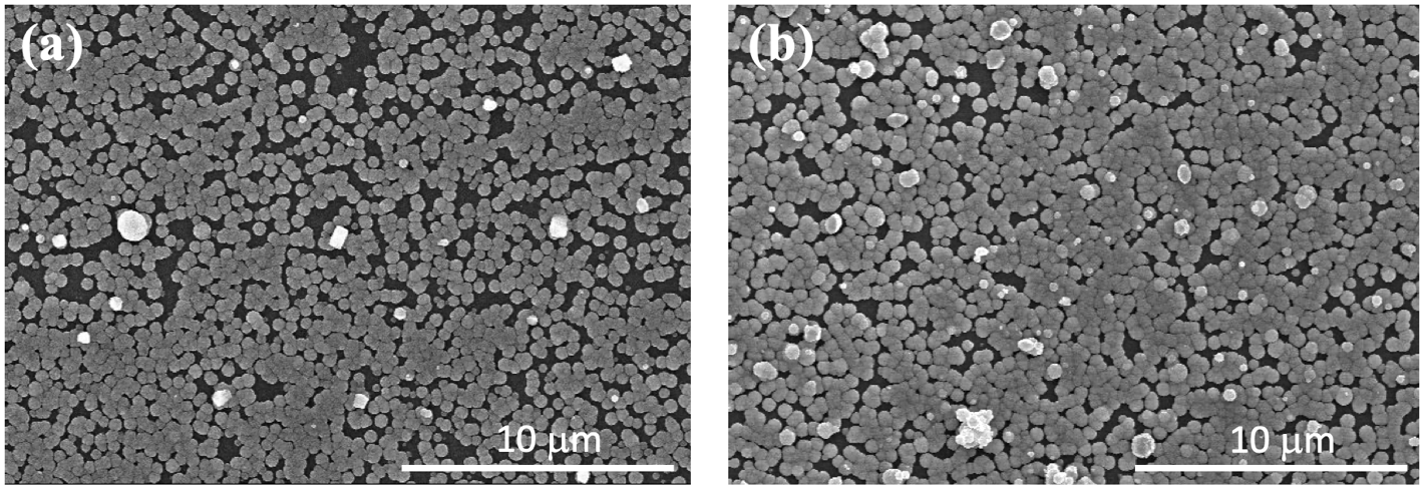


**Figure S6** Sb_2_S_3_ film morphological stability study via SEM. (a) SEM image of Sb_2_S_3_ film at 6 hrs CBD growth time before UV irradiation, (b) SEM image of the same Sb_2_S_3_ film from (a) after few months since UV-irradiation experiments
